# Society Meetings

**Published:** 1917-04

**Authors:** 


### SOCIETY MEETINGS

Brief reports and announcements of meetings of societies of Western New York. and those of general scope, are requested from Secretaries. Copy should be on hand the fifteenth of the month. Full transactions will be published at cost of composition.

---

**DOCTOR: Is your Society properly represented here? If not, please bring our standing announcement to the attention of the members.**

---

**The Elmira Academy of Medicine** held its regular meeting Mch. 5. Dr. A. H. Baker reported a case of Necrosis of Clavicle and Dr. G. M. Case read a paper on Recent Observations of Infections of Upper Air Passages.

---

**The Medical Society of the County of Chemung** held its regular meeting at Elmira, Mch. 20. Dr. Arthur W. Booth reported on the meeting of the council; Dr. Ross G. Loop presented some Comments on Recent Service at the Arnot-Ogden Memorial Hospital. Dr. A. H. Monroe read a paper, title not announced.

---

**The Rochester Academy of Medicine,** Section III, met Mch. 14. Dr. Joseph Roby reported on a Modification of Peypon Rouse's Test for Donor of Blood for Transfusion and Dr. Albert D. Kaiser read a paper on Serum and Blood Therapy.

---

**The Elmira Clinical Society** was entertained Mch. 16 by Dr. Charles Haase. Dr. C. F. Abbott gave a paper on Health Insurance and Dr. Haase on Fracture of the Arm.

---

The **Buffalo Academy of Medicine** has held the following meetings since last noted:

Section of Pathology: Feb. 28. "The Use of Renal Function in Cases of Nephritin," Dr. Henry A. Christian. Hersey Professor of the Theory and Practice of Physic,—Harvard Medical School. Physician-in-Chief, Peter Bent Brigham

Hospital, Boston, Mass. Dr. Allen A. Jones led the discussion. "The Study of the Effect of Tyroid Extract, on the Nitrogen Balance, Renal Function and Basal Metabolism in Cases of Chronic Nephritin and Hypo Thyroidism."* *This Study from Medical Clinic of the Peter Bent Brigham Hospital,—Dr. Byron D. Bowen, Buffalo.

Special meeting Mch. 2, Carrel Treatment of Wounds and Ambrine Treatment of Burns, illustrated by lantern slides, Dr. Wm. O'Neill Sherman of Pittsburgh.

Section of Surgery, Mch. 7, Diagnostic Value of Obstructive Jaundice, with lantern slides, Dr. Charles Gordon Hayd of Post Graduate Medical College, N. Y. Discussion led by Mrs. Edgar McGuire and Marshall Clinton.

Section of Medicine, Mch. 14, Poliomyelitis, Lessons from the Epidemic for the Clinician, Dr. Philip Van Ingen of N. Y. Discussion led by Dr. H. L. K. Shaw of Albany.

Obstetric Section, Mch. 21, Haemorrhages of Pregnancy, Including Placenta Praevia, Dr. Edward P. Davis, Philadelphia. Discussion led by Dr. Herman E. Hayd.

---

**The Medical Society of the County of Cattaraugus** met at Olean, Jan. 11. Officers were elected as follows: Pres., Dr. John C. Hoeffler of Salamanca; V. P. Dr. John A. Johnson of Olean; Sec.-Treas. Dr. H. H. Ashley of Machias.

